# Characteristics of Optic Disc Melanocytomas Presenting with Visual Dysfunction

**DOI:** 10.4103/0974-9233.65488

**Published:** 2010

**Authors:** Saba Al-Rashaed, Emad B. Abboud, Sawsan R. Nowilaty

**Affiliations:** Vitreoretinal Division, King Khaled Eye Specialist Hospital, Riyadh, Saudi Arabia

**Keywords:** Melanocytoma, Optic Disc, Visual Function

## Abstract

**Objective and Design:**

A retrospective review study was designed to describe five cases of optic disc melanocytomas with tumor-related visual impairment.

**Participants:**

Five patients with optic disc melanocytoma presented with visual complaints to a tertiary eye hospital in Saudi Arabia.

**Materials and Methods:**

Demographic and clinical data were analyzed, including the results of ocular examination, lesion laterality, best-corrected Snellen visual acuity, pupillary reflex, visual field testing, color fundus photography, fundus fluorescein angiography, and ophthalmic ultrasound.

**Results:**

Visual dysfunction secondary to optic disc melanocytoma was identified. Case 1 had macular star edema with mild tumor enlargement, Case 2 had optic atrophy, Case 3 had juxtapapillary choroidal neovascular membrane with macular involvement, Case 4 had optic disc swelling with an enlarged blind spot, and Case 5 had a large altitudinal visual field defect.

**Conclusion:**

Although melanocytomas of the optic disc tend to have a benign behavior with slow evolution and stable vision, they may adversely affect visual function through a variety of mechanisms.

## INTRODUCTION

Melanocytoma, also known as *Magnocellular nevus*, is a benign, stationary heavily pigmented tumor that may develop wherever uveal melanocytes are present and most commonly occur on, or, adjacent to the optic with little potential for growth.^1^,^2^

Frequently melanocytomas are identified coincidentally, and visual impairment, apart from an enlarged blind spot when the tumor occupies the optic disc, is rare. However, significant visual loss has been reported.^3^ Instances where melanocytomas enlarge rapidly and lead to sudden visual loss and necrosis can be found in the literature.^2^–^4^

Rare cases of melanomas emerging from melanocytomas have also been reported.^5^–^7^ Shields *et al*. recently documented that the rate of malignant transformation is approximately 1–2%.^4^

In this case study, we present five interesting cases of optic disc melanocytomas with visual complaints that were attributed to the tumor.

## MATERIALS AND METHODS

**Figure 1:**
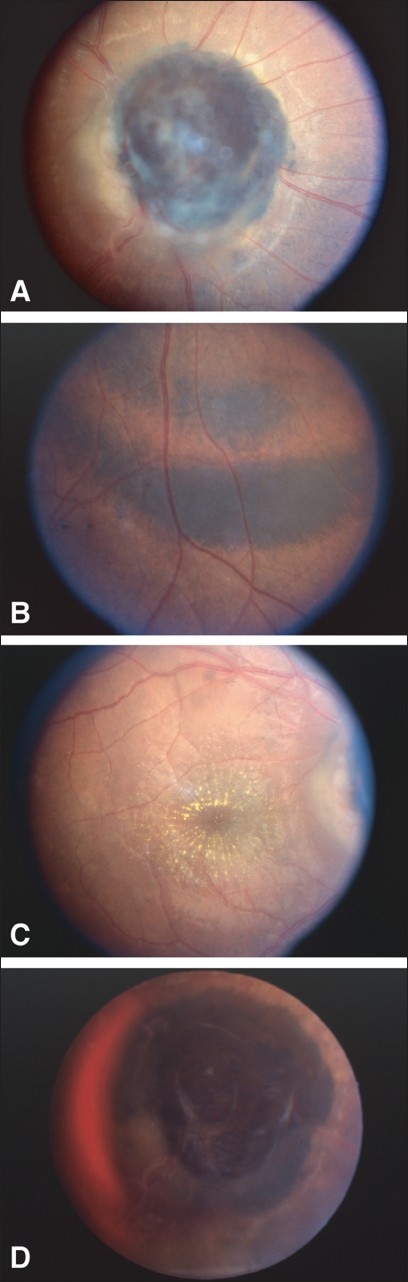
Case 1 (A) Optic disc melanocytoma, (B) pigment dispersion from tumor surface to peripheral fundus, (C) macular star, and (D) tumor enlargement after 2 years follow-up

**Case 1 [Figure 1]:** A 16-year-old woman presented with gradual decrease in visual acuity in the right eye over 2 months. Visual acuity (VA) in her right and left eyes was 20/80 and 20/20, respectively. Pupillary examination was positive for an afferent pupillary defect (APD). Funduscopic examination revealed the presence of a jet-black tumor on the optic disc with exudation and subretinal fluid around the tumor and secondary macular star edema. Pigmentary dispersion with vitreous seeds was present in the entire fundus. The tumor was diagnosed as an optic disc melanocytoma. Intravenous fluorescein angiography (IVFA) findings included, blockage at the site of tumor and leaking around the tumor with a window defect corresponding to the retinal pigment epithelium (RPE) mottling the macular area. A highly reflective irregular nonvascular structure was imaged with A scan ultrasonography. The basal diameter was 5.3 mm, and tumor thickness was 5.3 mm. The patient was followed up for 2 years and on her last visit, the tumor was mildly enlarged (0.8 mm) in basal diameter, and there was greater pigmentary dispersion in the fundus and vitreous seeds. However, her visual acuity remained unchanged.

**Figure 2:**
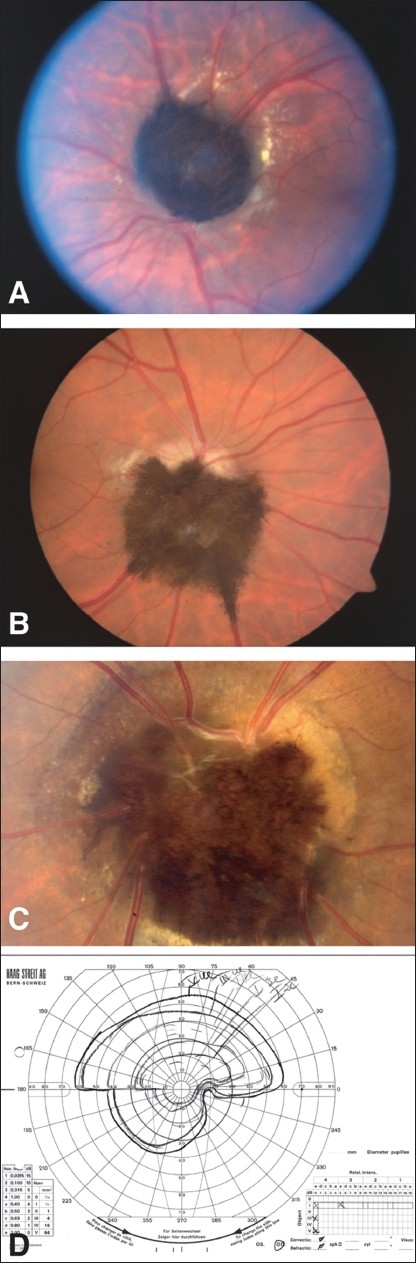
Case 2 (A) Optic disc melanocytoma with ischemic optic neuropathy, (B) patient had visual improvement after 6 years of follow-up. Note the direction of the tumor’s growth has also changed. Case 5 (C and D) Inferior optic disc melanocytoma and corresponding altitudinal visual field defect

**Case 2 [Figure 2A, B]:** A 30-year-old man presented with sudden loss of vision in the right eye. VA in the right eye was hand motion and 20/20 in the left eye. An APD was present. Funduscopic examination revealed a jet-black tumor encompassing the entire optic disc with feathery changes in the retina, pigment dispersion in the retina and exudates with chronic subretinal fluid (SRF) surrounding the tumor. Ophthalmic ultrasound (A and B scans) findings indicated a highly reflective dome-shaped tumor. The basal diameter was noted to be 3.2 mm, and tumor thickness was 3.5 mm. IVFA revealed a blockage at the site of tumor; however, there were no signs of vascular occlusion. Ocular Doppler ultrasound examination was performed to exclude vascular occlusion, and it was within normal limits. Only a temporal islands of vision were present on visual field testing. On the basis of the above findings, the patient was diagnosed with optic atrophy resulting from compression of the optic nerve fibers by the tumor. The patient returned for follow-up 3 year after initial presentation. Changes in the direction of the tumor allowed visualization of a pale optic disc on funduscopy 3 years after initial presentation. VA improved from HM to 2/200; however, there was no change in visual field and tumor size 3 years after initial presentation.

**Figure 3:**
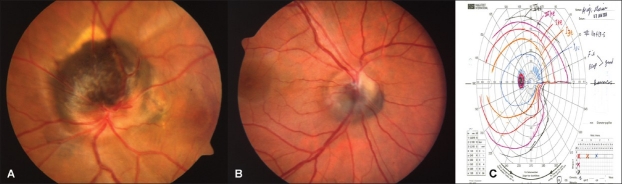
Case 3 (A) Optic disc melanocytoma with adjacent involutedly CNVM with secondary pigmentary changes in the fovea. Case 4 (B and C) Inferior optic disc melanocytoma with optic disc swelling and corresponding visual field infranasal step

**Case 3 [Figure 3A]:** A 40-year-old woman presented with history of gradual decrease in vision in the left eye and metamorphopsia for over a 3-month period. The VA was 20/20 in the right eye and 20/70 in left eye. A jet-black tumor on the optic disc with an adjacent involuted choriodal neovascular membrane (CNVM) extending subfoveally with secondary postexudative pigmentary changes in the fovea were present on funduscopy. IVFA documented blockage at the site of the tumor with staining of adjacent area that corresponded to the area of involuted CNVM and window defect in the macular area. The patient was diagnosed with optic disc melanocytoma associated with juxta-papillary CNVM. The patient did not return for a follow-up visit.

**Case 4 [Figure 3B and C]:** A 36-year-old woman with a history of headache and blurry vision was referred for suspected papilledema. VA was 20/25 in both eyes. Funduscopic examination revealed an inferior jet-black optic disc melanocytoma with feathery extension into the retina and optic disc swelling in the left eye and APD. The optic disc in the other eye was normal. The visual field examination revealed enlargement of blind spot and an inferonasal step. Elements of mild optic neuropathy could not be excluded at the time of presentation. Hence, the patient underwent orbital and brain computed tomography (CT) scans, magnetic resonance imaging (MRI), and lumbar puncture to exclude benign intracranial hypertension and space occupying lesion. These tests were normal except for CT scan which indicated sinusitis. Ophthalmic ultrasound (A and B scans) findings indicated a solid, medium to highly reflective tumor. The basal diameter was 2.2 mm, and tumor thickness was 1.7 mm. This patent was followed up for over 20 years, and her condition remained stable.

**Case 5 [Figure 2C and 2D]:** A 39-year-old man presented with a history of blurred vision in the right eye for 2 years. Visual acuity was 20/20 in both eyes. Funduscopic examination revealed a jet-black tumor inferior to the optic disc with feathery extension into the retina, pigment dispersion in the retina, and vitreous seeds. There was SRF around the tumor. Ophthalmic ultrasound (A and B scans) indicated a highly reflective tumor, with a basal diameter of 4.0 mm and tumor thickness of 2.8 mm. The pupil reaction was normal; however, the VF test exhibited an altitudinal field defect and enlargement of the blind spot. The patient was diagnosed with optic disc compression due to optic disc melanocytoma. Four months after presentation, there were no significant changes noted. The patient was lost to follow up.

## DISCUSSION

Melanocytoma consists of a special type of nevus and can occur anywhere that these cells are present including the iris, ciliary body, and the optic disc. The lamina cribrosa of the optic nerve head, with its population of melanocytes, is the origin of these pigmented lesions on the optic nerve.^2^

Melanocytomas appear as gray to jet-back elevated lesions and usually do not exceed one disc diameter in size on fundus examination. However, one report presented a lesion that was six disk diameters in size.^8^ Melanocytomas are usually asymptomatic. A slow impairment of visual acuity attributed to the tumor is reported to occur in approximately 5% of cases. Cases accompanied by significant loss of visual acuity and/or visual field have also been reported.^3^,^9^–^12^ Similarly, in our cases, tumor-related visual impairment was due to macular exudation (Case 1), optic atrophy from optic nerve compression by the tumor (Case 2), and foveal choroidal neovascularization (Case 3). Cases 4 and 5 demonstrated blurred vision, visual field defects, and clinical examination revealed optic disc edema in Case 4 and peripapillary subretinal fluid in Case 5.

In conclusion, although optic disc melanocytomas tend to be benign behavior, they may adversely affect visual function.
